# Inactivation of glycogen synthase kinase-3α is required for mitochondria-mediated apoptotic germ cell phagocytosis in Sertoli cells

**DOI:** 10.18632/aging.101614

**Published:** 2018-11-06

**Authors:** Yabin Gong, Zhilong Zhang, Zhanglin Chang, Hao Zhou, Ruqian Zhao, Bin He

**Affiliations:** 1 Key Laboratory of Animal Physiology and Biochemistry, College of Veterinary Medicine, Nanjing Agricultural University, Nanjing 210095, PR China; 2 MOE Joint International Research Laboratory of Animal Health and Food Safety, Nanjing Agricultural University, Nanjing 210095, PR China; 3 Jiangsu Collaborative Innovation Centre of Meat Production and Processing, Quality and Safety Control, Nanjing 210095, PR China; * Equal contribution

**Keywords:** glycogen synthase kinase-3α, phagocytosis, mitochondrial fission, Sertoli cell, spermatogenesis

## Abstract

The rapid and efficient clearance of apoptotic germ cells (GCs) by Sertoli cells (SCs) is important for spermatogenesis. High mitochondrial activity in phagocytes is critical for continued clearance of apoptotic cells. However, the underlying molecular mechanism is poorly understood. Glycogen synthase kinase-3α (GSK3α) is a protein kinase that participates in the regulation of mitochondrial activity. Immunohistochemistry evidenced the predominant presence of the Ser21 phosphorylation GSK3α (inactivation) signal in SCs. Heat shock-induced apoptosis of GCs and dephosphorylation of GSK3α in SCs is a perfect model to investigate the role of GSK3α in phagocytic action. The number of apoptotic GCs was significantly lower in GSK3α inhibitor pre-treated mice with HS compared to normal control. *In vitro* phagocytosis assays shown that the phagocytic activity in GSK3α activated SCs was downregulated, while GSK3α inhibitor supplementation restored this process. Moreover, GSK3α activation participates in the alteration of the mitochondrial ultrastructure and activity. In particular, GSK3α activation inhibits mitochondrial fission via phosphorylation of dynamin related protein 1 at Ser637. Changes of mitochondrial activity resulted in the accumulation of lipid droplets and the alteration of metabolism pattern in SCs. In summary, our results demonstrate that inactivation of GSK3α is required for mitochondria-mediated apoptotic GCs phagocytosis in SCs.

## Introduction

During mammalian spermatogenesis, more than 75% of the developing spermatogenic cells undergo apoptosis before maturation [1]. Apoptotic spermatogenic cells can provide energy sources for Sertoli cells (SCs) [2]. The rapid and efficient degradation of apoptotic germ cells (GCs) by SCs has been suggested as crucial for appropriate germ cell development and differentiation. Impaired SC phagocytosis can lead to noninfectious inflammatory responses in the testis [3]. Although the occurrence of apoptosis at various stages of spermatogenesis has been observed frequently, few apoptotic GCs are histochemical detectable in normal adult testis. This is most probably due to their rapid elimination via phagocytosis. This phagocytic action is necessary for the maintenance of testicular homeostasis under both physiological and pathological conditions. Although the investigation of these phenomena has recently become more intensive, most mechanisms still remain unclear.

Several molecules including the Dock180-Elmo1-Rac1 signaling network are α-taxilin protein/ATP-binding cassette transporter 1 (TXLNA/ABCA1) cascade and are involved in the engulfment of apoptotic GCs by SCs [4,5]. After apoptotic GCs are engulfed, the lysosome-dependent clearance process begins in the SCs. It has been reported that apoptotic GCs and residual bodies can be used to produce ATP by SCs after their phagocytosis [2]. Mitochondrial metabolic activity in phagocytes is critical for continued clearance of apoptotic cells [6,7]. Park et al. indicated that both the mitochondrial membrane potential (ΔΨm) and uncoupling protein 2 (Ucp2) are key molecular determinants of apoptotic cell clearance [6]. The testis of mice with Ucp2 mutation mice contained increased numbers of uncleaned apoptotic cells compared to Ucp2 wild type mice [6]. Recently, Wang et al. demonstrated that the uptake of multiple apoptotic cells by macrophages requires dynamin related protein 1 (Drp1)-mediated mitochondrial fission [7]. However, the regulation of the mitochondrial fission, mitochondrial activity, and mitochondria-mediated apoptotic GCs phagocytosis in SCs have not been clarified to date.

Glycogen synthase kinase-3 (GSK3) is a serine/ threonine protein kinase that mediates a large number of cellular processes. It is encoded by two genes that generate two related proteins: GSK3α and GSK3β [8]. GSK3α can be inactivated via Ser21 phosphorylation. GSK3α expression increases during the onset of spermatogenesis and reaches a maximum in adult testis. Targeted disruption of GSK3α in mice affects sperm motility, which results in male infertility [9,10]. GSK3-induced mitochondrial fragmentation through phosphorylating Drp1 at Ser40 and Ser44 residues was crucial for the pathogenesis of Alzheimer’s disease [11]. The phosphorylation of Drp1 at Ser637 could inhibit Drp1 translocation to mitochondria and thus promote mitochondrial fusion [12]. It is plausible that GSK3α may contribute to mitochondrial activity and mitochondria-mediated apoptotic GCs phagocytosis in SCs through a Drp1-dependent mechanism.

Experimentally, forced heat shock (HS) on the testis causes GC apoptosis, leading to subfertility or even infertility. Our previous results demonstrated that HS induced Ser21 dephosphorylation of GSK3α [13]. Therefore, HS-induced apoptosis of GCs in testis and activation of GSK3α in SCs are perfect models for the investigation of the regulation of GSK3α in phagocytic action. The obtained results will help to delineate the molecular mechanisms involved in mitochondria-mediated apoptotic GCs phagocytosis in SCs.

## RESULTS

### Distinct pattern of GSK3α phosphorylation in mouse SCs

**Figure 1:**
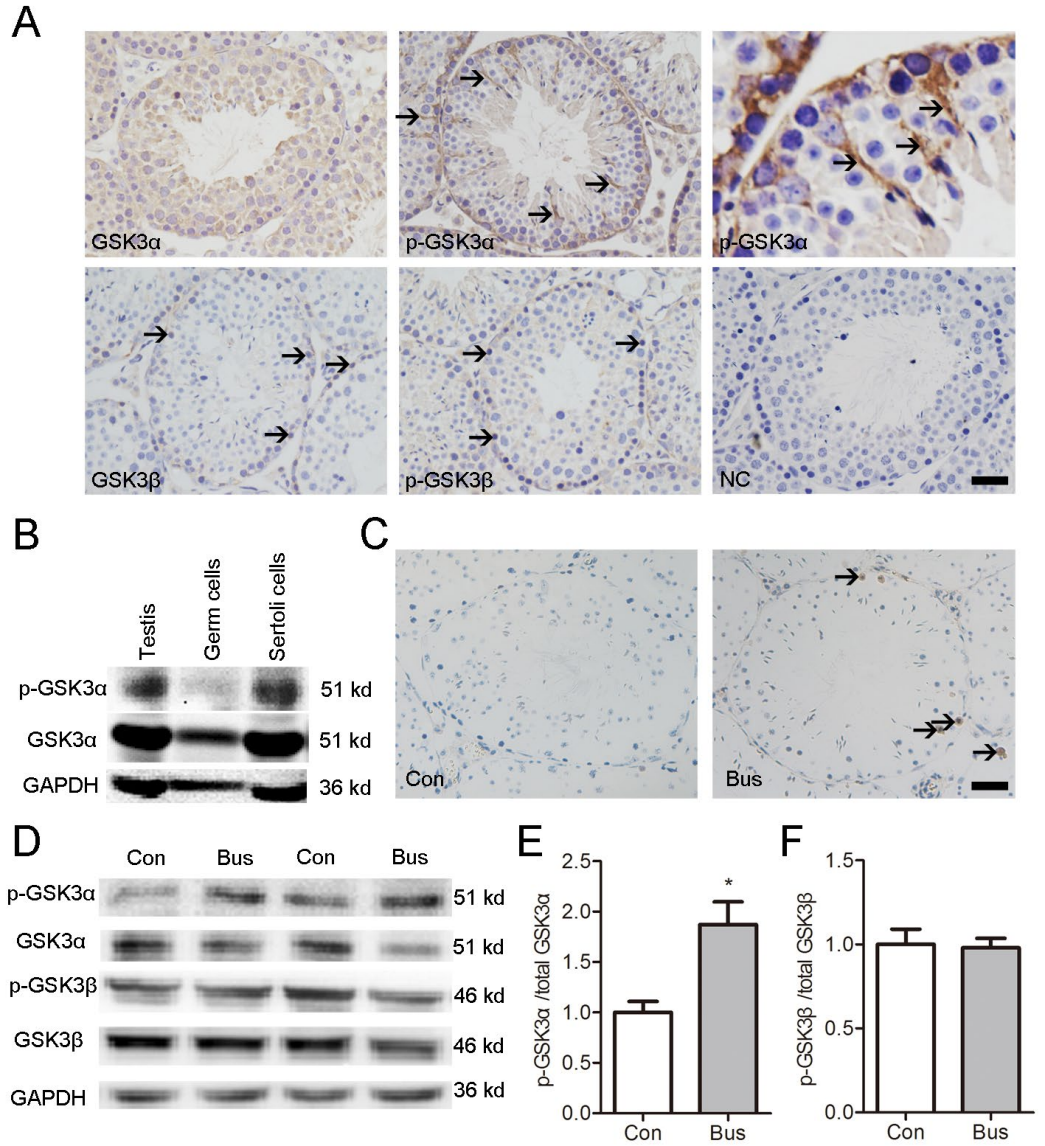
**Localization of p-GSK3α in mouse testis and association between apoptotic germ cells and p-GSK3α in Sertoli cells.** (**A**) Representative microscopic images of GSK3α, p-GSK3α, GSK3β, and p-GSK3β in mouse testis evaluated by immunohistochemistry. (**B**) Protein levels of GSK3α and p-GSK3α in germ cells and Sertoli cells evaluated by western blot. (**C**) TUNEL staining of testicular sections were carried out at 14 d after busulfan treatment. Brown nuclear staining indicates apoptotic cells (arrow). (**D**) Western blots showing the protein levels of p-GSK3α, total GSK3α, p-GSK3β and total GSK3β in testis of adult mice after busulfan treatment for 14 d. (**E**) Histogram indicates the ratio of p-GSK3α/GSK3α. (**F**) Histogram indicates the ratio of p-GSK3β/GSK3β. Con, control; Bus, busulfan. Scale bars = 50 μm. Values are expressed as the mean±SEM, n=6; * *P* < 0.05.

Immunohistochemistry test results indicated that GSK3α is ubiquitously and abundantly located in mouse seminiferous tubules, without discernible stage or cell type specificity (Fig. 1A). However, immunohisto-chemistry evidenced the predominant presence of Ser21 phosphorylation of the GSK3α signal in the SCs (Fig. 1A). Western blot results indicated that GSK3α can be detected in GCs, SCs, testis and caudal sperm. However, p-GSK3α were detected in Seroli cells, testis and caudal sperm but not in GCs (Fig. 1B). In contrast to GSK3α, GSK3β can be found via cytoplasmic immunostaining in spermatogonia and preleptotene spermatocytes (Fig. 1D). Furthermore, Ser9 p-GSK3β can be found via cytoplasmic immunostaining in spermatogonia and preleptotene spermatocytes (Fig. 1A).

To elucidate the effect of apoptotic GCs on the Ser21 phosphorylation of GSK3α, we treated mice were treated with busulfan. In line with a previous report, massive apoptotic spermatogenic cells appeared in busulfan treated testis at posttreatment d14 (Fig 1C). The level of p-GSK3α (Ser21) and ratio of p-GSK3α/total GSK3α were higher in busulfan treated testis compared to control (Fig. 1D-E). Moreover, no significant difference was found in the levels of p-GSK3β and total GSK3β as well as the rate of p-GSK3β/GSK3β between groups (Fig. 1D, F). In summary, these data collectively suggest an association between apoptotic GCs and Ser21 phosphorylation GSK3α in SCs.

### GSK3α inactivation is required for apoptotic GCs clearance

**Figure 2:**
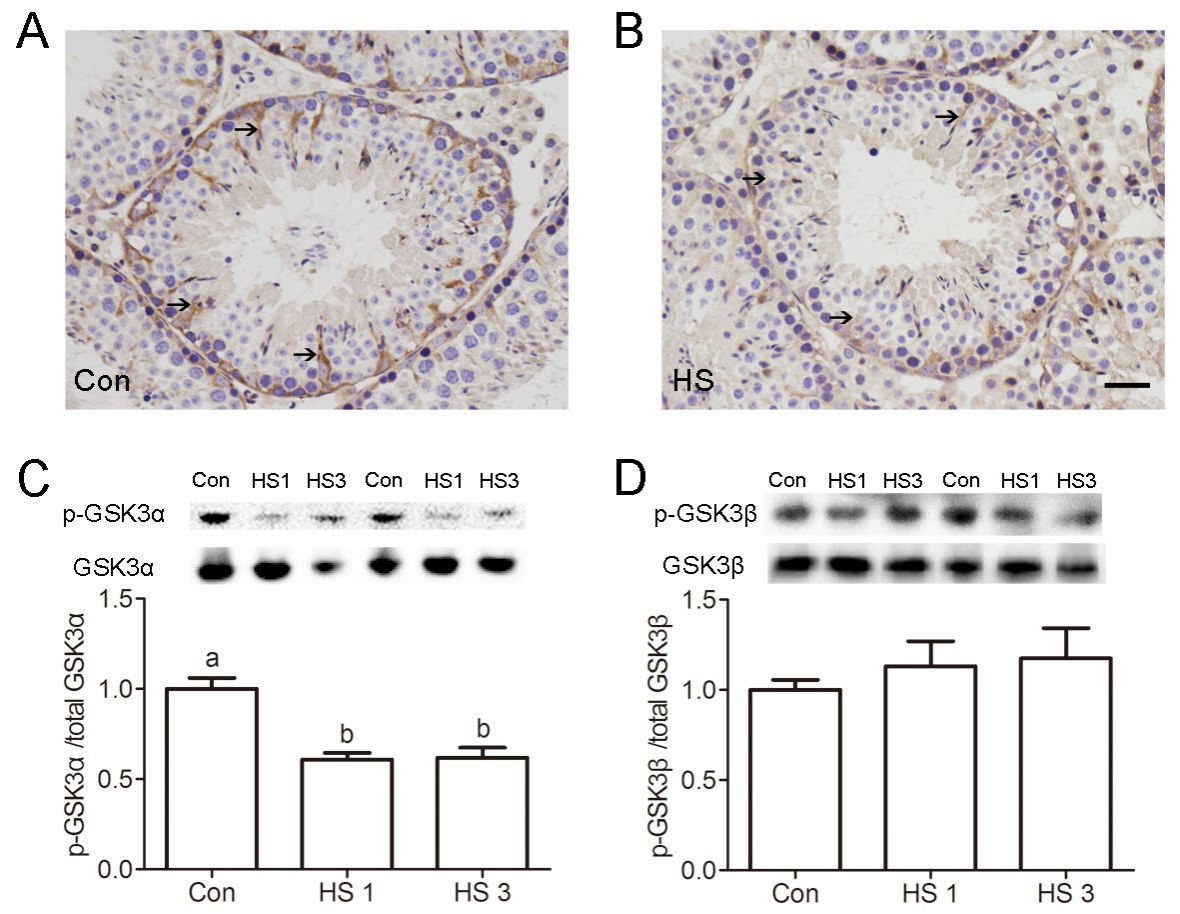
**Heat shock-induced dephosphorylation of GSK3α in Sertoli cells.** (**A-B**) Representative microscopic images of p-GSK3α in control (**A**) and heat shock (HS) treated (**B**) mouse testis evaluated by immunohistochemistry. Arrows indicate p-GSK3α-positive spermatocytes. Scale bar=50 μm. (**C**) Western blots and histogram showing the protein levels of p-GSK3α and GSK3α in mouse testis after heat shock. (**D**) Western blots and histogram showing the protein levels of p-GSK3β and GSK3β in mouse testis after heat shock. Con, control; HS, heat shock. Values are expressed as the mean±SEM, n=6. Values with different superscripts are significantly different from each other (*P*<0.05).

Then, a mouse model we developed to investigate whether GSK3α inactivation is required for the clearance of apoptotic GCs. After a single, mild, transient scrotal HS, p-GSK3α (Ser21) was found to have decreased in SCs was reduced (Fig. 2A-B). Furthermore, the levels of p-GSK3α (Ser21) and total GSK3α were detected via Western blot. The levels of p-GSK3α (Ser21) were lower in HS-treated testis compared to control (Fig. 2C). There was no significant difference in levels of p-GSK3β (Ser9) and total GSK3β as well as in the rate of p-GSK3β/GSK3β between groups (Fig. 2D).

**Figure 3:**
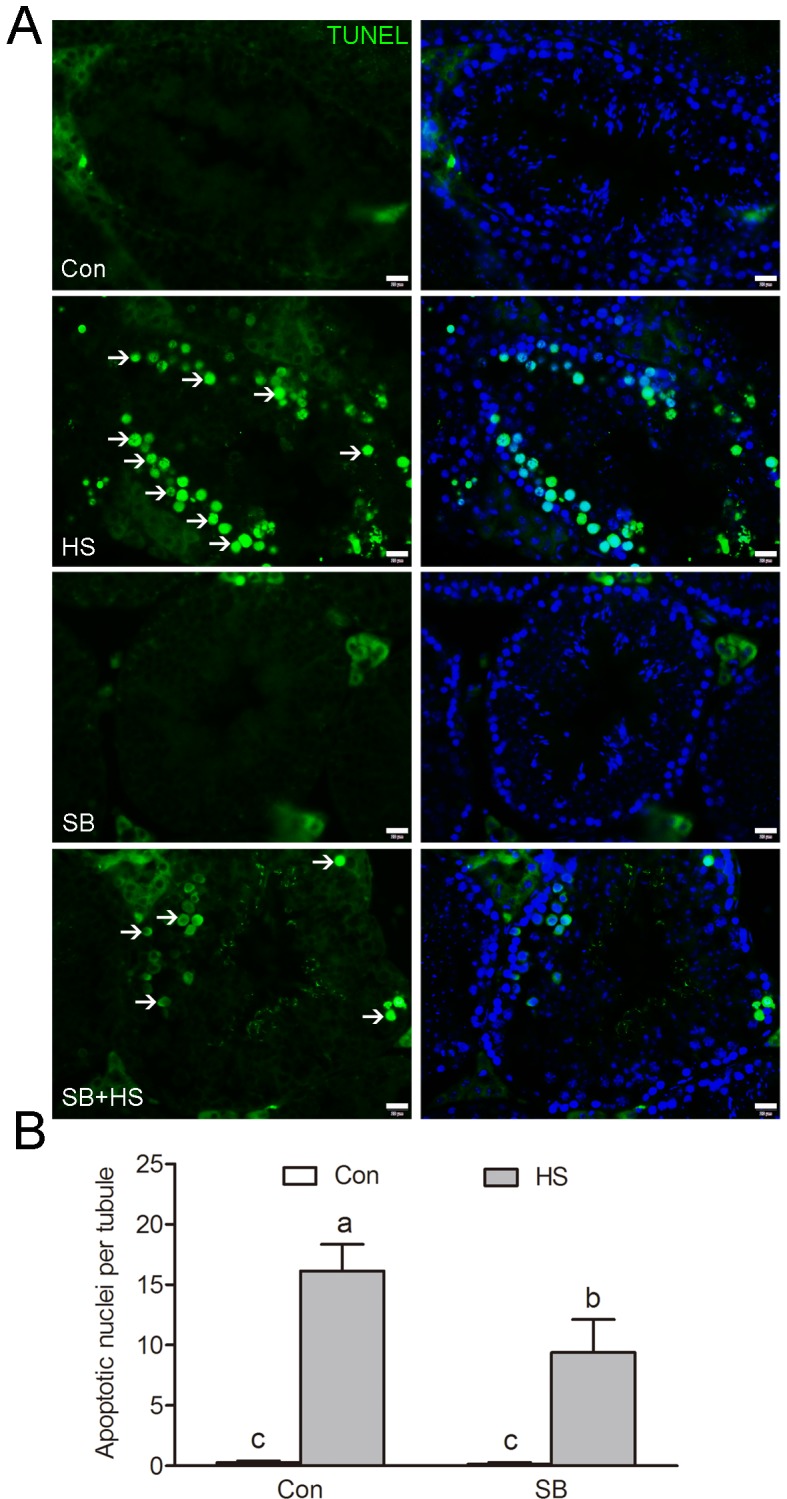
**GSK3α inhibition is required for apoptotic GCs clearance.** (**A**) Apoptotic cells revealed by TUNEL assay on testis sections. Representative seminiferous tubules are shown. The green signal (arrow) indicates a TUNEL-positive nucleus. Con: control; HS: heat shock; SB: SB216763. Scale bar=20 μm. (**B**) Histogram showing the average number of TUNEL-positive cells per seminiferous tubule. Values are expressed as the mean ± SEM, n=15. Values with different superscripts are significantly different from each other (*P*<0.05).

Efficient phagocytic clearance of apoptotic GCs by adjacent SCs is central for functionally mature spermatogenesis. To test the effects of GSK3α ablation on testicular phagocytic activity, mice were treated with a GSK3α inhibitor, SB216763 through the caudal vein. Histological examinations shows a significantly lower number of apoptotic germ cell nuclei in SB216763 treated mice compared to HS-treated mice (Fig. 3A-B). In summary, these data confirmed the potential that GSK3α inactivation was associated with testicular phagocytosis.

### GSK3α inactivation participates in phagocytosis

**Figure 4:**
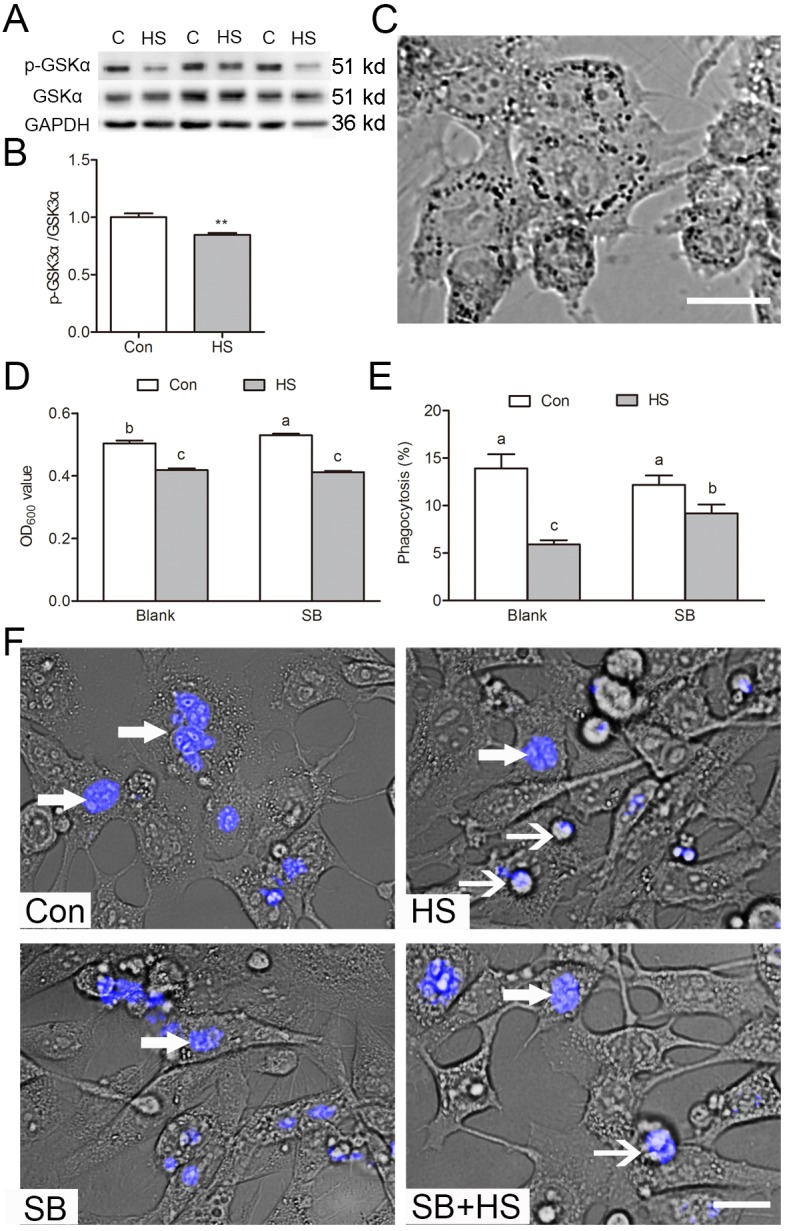
**GSK3α participates in Sertoli cell phagocytosis of apoptotic germ cells.** (**A-B**) Western blots and histogram showing the protein levels of GSK3α and p-GSK3α in control and HS treated TM4 cells. C: control; HS: heat shock. (**C**) The phagocytosis of Indian ink beads by TM4 cells observed by light microscopy. Black dots indicate engulfed Indian ink in TM4 cells. (**D**) Quantification of Indian ink beads phagocytosis via Micro plate spectrophotometer read as an OD value. (**E**) Histogram showing percentage of TM4 cells engulfing apoptotic germ cells as derived from immunofluorescence analysis. (**F**) Immunofluorescence analysis showing phagocytosis of apoptotic germ cells by TM4 cells treated with HS or GSK3α inhibitor. TM4 cells were fed with apoptotic male germ cells labeled with DAPI. Con: control, HS: heat shock, SB: SB216763. Thick arrow indicates engulfed germ cells. Thin arrow indicates unengulfed germ cells. Scale bars=10 μm. Values are expressed as the mean±SEM, n=15. Values with different superscripts are significantly different from each other (*P*<0.05).

To investigate whether the inactivation of GSK3α affected the ability of SCs to engulf apoptotic GCs, *in vitro* phagocytosis assays were performed with TM4 Sertoli cells. A decrease of Ser21 phosphorylation of GSK3α after 3 h heat treatment in TM4 cells was observed (Fig. 4A-B). Indian ink analysis shown that phagocytic activity of TM4 cells was decreased after GSK3α activation (Fig. 4C-D). However, it cannot be rescued by GSK3α inhibitor co-treatment. To further substantiate these findings, a phagocytosis assay was performed in both presence and absence of apoptotic GCs. The phagocytosis in GSK3α activated SCs were decreased, whereas GSK3α inhibitor supplementation restored the level of phagocytosis (Fig. 4E-F). In summary, these results indicated that GSK3α is required for apoptotic GCs phagocytosis in SCs.

### GSK3α activation inhibits mitochondrial fission via phosphorylation of Drp1 at Ser637

**Figure 5:**
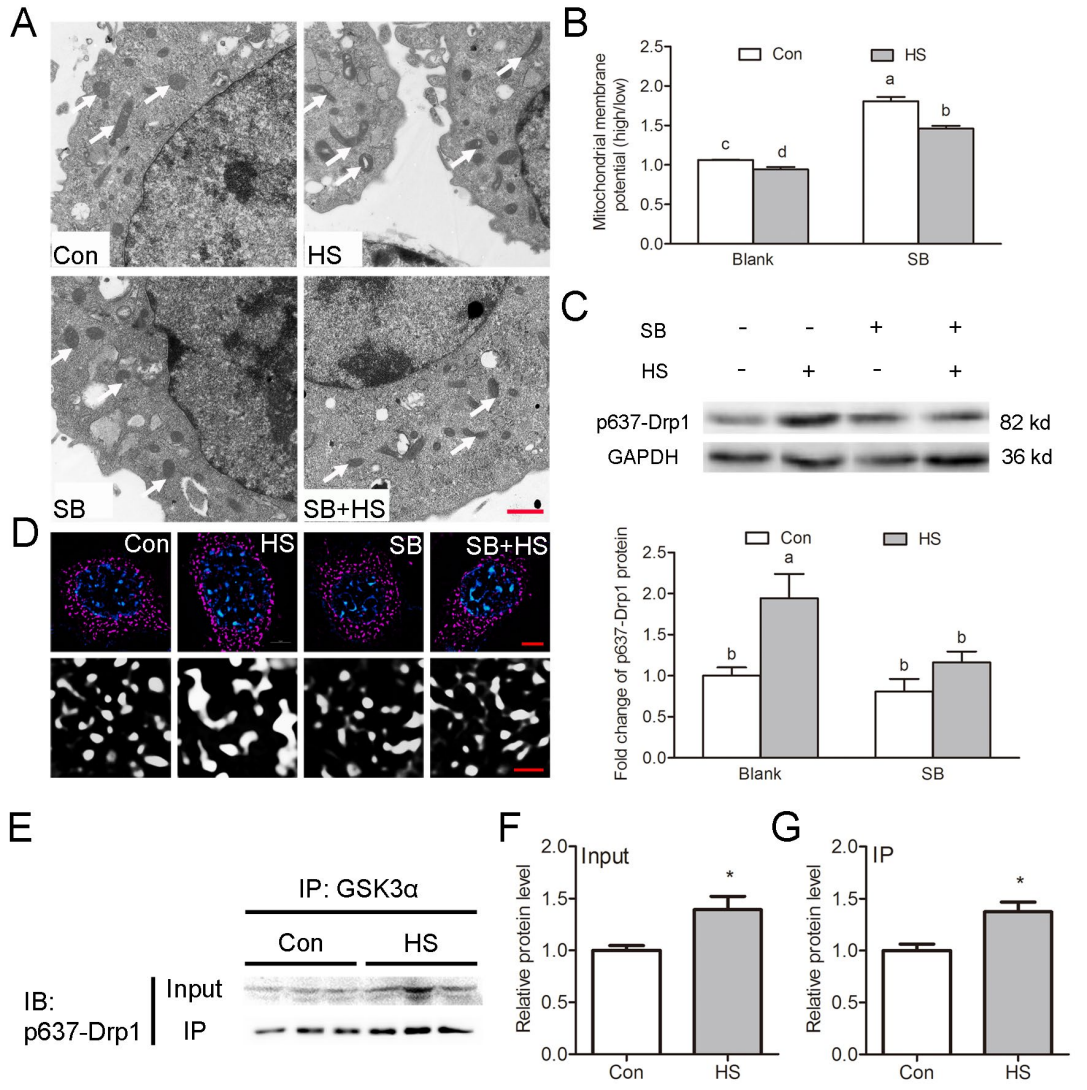
**GSK3α activation inhibits mitochondrial fission through phosphorylation of Drp1 at Ser637 in Sertoli cells.** (**A**) Representative electron microscopic images of mitochondrial ultrastructure in TM4 cells treated with HS or GSK3α inhibitor. Arrows indicate mitochondria. Scale bars=1 μm. (**B**) Histogram showing ΔΨm in TM4 cells by JC-1 staining and flow cytometric analysis (**C**) Western blots and histogram showing the protein levels of Ser637-Drp1 in TM4 cells treated with HS or GSK3α inhibitor. (**D**) Representative immunofluorescence images of mitochondria (red) in TM4 cells treated with HS or GSK3α inhibitor. (**E-G**) Immunoprecipitation of Ser637-Drp1 with anti-GSK3α in TM4 cell followed by immunoblotting analysis to demonstrate the stimulatory effects of HS on the interaction between GSK3α and Ser637-Drp1. Con: control; HS: heat shock; SB: SB216763. Scale bars=1 μm. Values are expressed as the mean±SEM, n=6. Values with different superscripts are significantly different from each other (*P*<0.05).

Transmission electron microscopy was employed to evaluate the mitochondrial ultrastructure in TM4 cells. Most of the mitochondria contain clearly visible intact inner membrane, outer membrane, and a well-defined inter membrane space (Fig. 5A). In contrast, marked ultrastructural changes in the mitochondria including disorientation, swelling, and vacuole structures were observed in heat-treated cells (Fig. 5B). Treatment with the GSK3α inhibitor SB216763 reversed the HS-induced damage of the mitochondrial ultrastructure in TM4 cells. The mitochondrial probe JC-1 was used to assess ΔΨm in TM4 cells. The number of high ΔΨm was significantly lower in HS than in the control group (Fig. 5B). SB216763 treatment significantly reversed the HS-induced decrease of ΔΨm (Fig. 5B).

Morphological examination showed that mitochondria in HS-treated cell were more longer while control cells maintained predominantly round mitochondria (Fig.5A, D). Recently, Wang et al. demonstrated that the uptake of multiple apoptotic cells by macrophages requires Drp1-mediated mitochondrial fission [7]. Phosphorylation of Drp1 at Ser637 could inhibit the translocation of Drp1 to mitochondria and thus block mitochondrial fission [12]. Here, Drp1 phosphorylation at Ser637 was observed to be significantly increased in GSK3α activated TM4 cells (Fig. 5C). Moreover, the increase of phosphorylation at Ser637 induced by HS can be blocked by SB216763 (Fig. 5C). Co-immunoprecipitation analysis showed that GSK3α interacted with Ser637-Drp1 (Fig. 5E-G). In summary, these results indicated that GSK3α activation inhibits mitochondrial fission via phosphorylation of Drp1 at Ser637.

### GSK3α activation participates in lipid droplet accumulation

**Figure 6:**
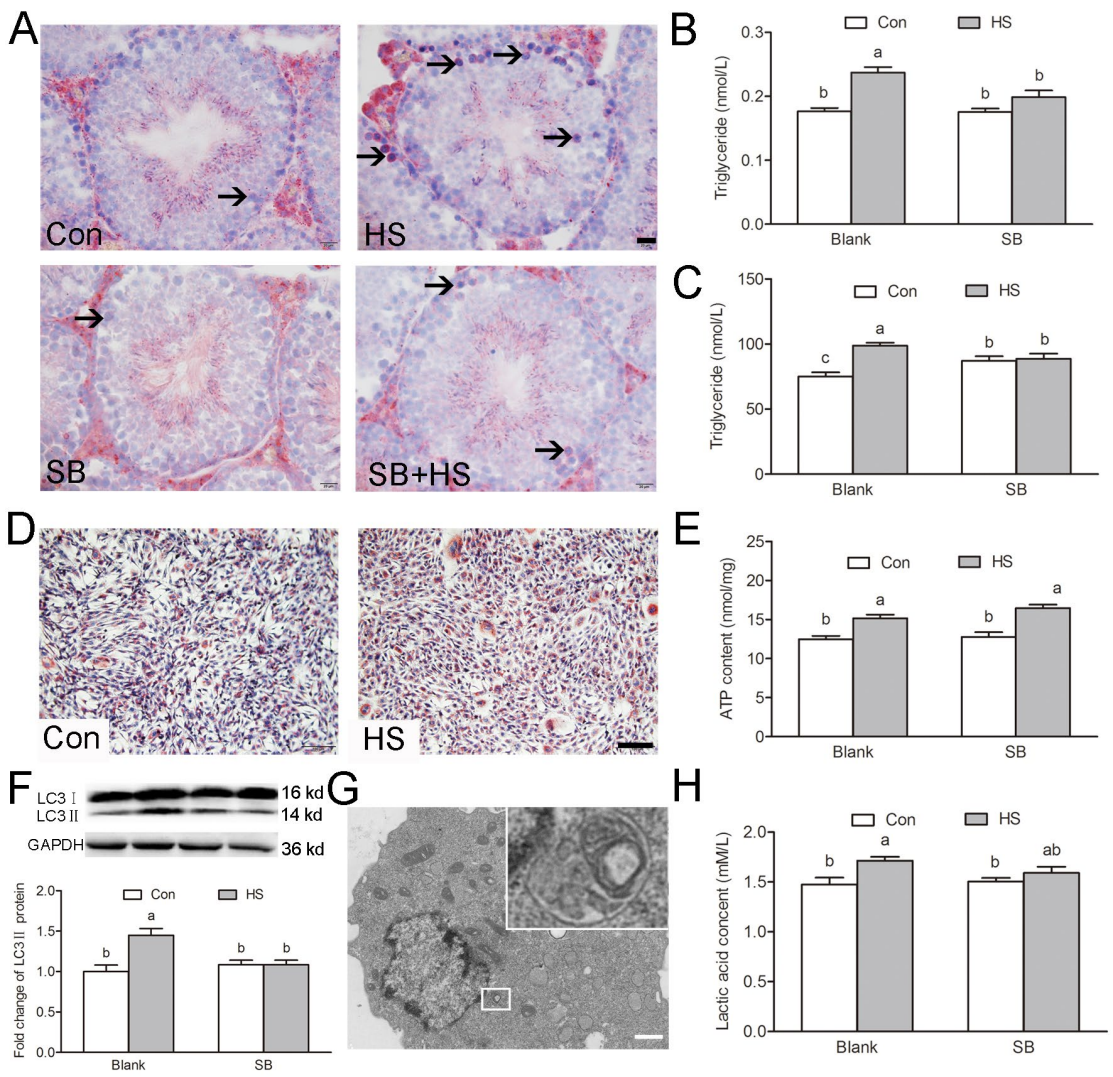
**GSK3α activation participates in HS-induced Sertoli cells lipid droplets accumulation.** (**A**) Representative microscopic images of lipid droplet formation in mouse testis treated with HS or GSK3α inhibitor. ORO stained lipid droplets are shown in red (arrows). Scale bars=20 μm. (**B**) Histogram showing quantification of TG content in mouse testis treated with HS or GSK3α inhibitor. (**C**) Histogram showing quantification of TG content in TM4 cells treated with HS or GSK3α inhibitor. (**D**) Representative microscopic images of lipid droplet formation in TM4 cells. Scale bars=50 μm. (**E**) Histogram showing quantification of ATP content in TM4 cells treated with HS or GSK3α inhibitor. (**F**) Western blots and histogram showing the protein levels of LC3 in TM4 cells. (**G**) Representative electron microscopic images of autophagosome structure in TM4 cells. (**H**) Histogram showing quantification of lactic acid content in TM4 cells treated with HS or GSK3α inhibitor. Con: control; HS: heat shock; SB: SB216763. Values are expressed as the mean±SEM, n=6. Values with different superscripts are significantly different from each other (*P*<0.05).

Phagocytosis of apoptotic GCs results in the formation of lipids, which are further metabolized to produce ATP in SCs. The deregulated metabolism of lipid droplets is the hallmark of disrupted SC function [2]. A population of small lipid droplets, located in the area of the epithelium faces, the lumina of most of the tubules (i.e., mostly extracellular) after HS (Fig. 6A). Biochemical tests confirmed the increase of the triglyceride content in the testis (Fig. 6B). *In vitro* studies found that GSK3α activation resulted in significant increases of triglyceride content (Fig. 6C) and lipid droplets in TM4 cells (Fig. 6D).

LC3-associated phagocytosis (LAP) has been reported to play an equally important role in the clearance of phagocytosed apoptotic germ cells in SCs [14]. The LC3 conversion (LC3I to LC3II) is clearly correlated with the autophagy, therefore the increased levels of LC3II are important markers for enhanced autophagy [15]. Increase of LC3II was observed in TM4 cells in response to HS (Fig. 6F-G). However, GSK3α inhibitor supplementation blocked the augment of autophagy. These results indicated that GSK3α activation participates in autophagy but not LAP in TM4 cells.

The total ATP content was increased after HS suggesting that HS changed the energy metabolism pattern in SCs (Fig. 6E). Previous studies have shown that HS-induced autophagy participated in the lactate secretion in SCs [16]. Our results show that the lactic acid content increased significantly after HS (Fig. 6H). These phenomena can be blocked by a GSK3α inhibitor, which confirms that GSK3α activation is involved in the alteration of the energy metabolism pattern in SCs. GSK3α activation induced the β-oxidation disorder in testicles and led to the accumulation of lipid droplets, while releasing a large quantity of lactic acid through glycolysis in SCs.

## DISCUSSION

**Figure 7:**
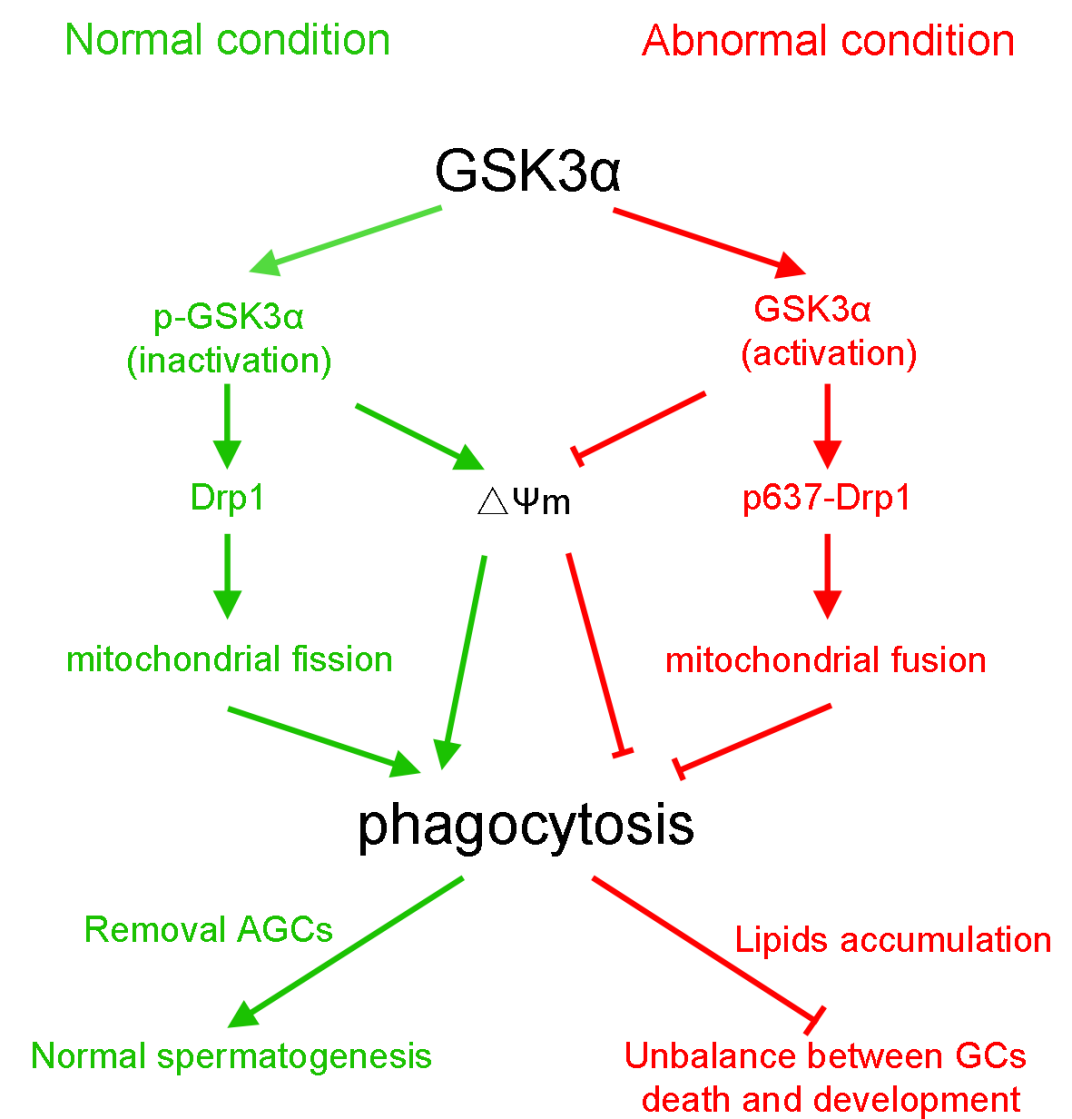
The schematic diagram demonstrates intracellular signaling events in GSK3α participates in mitochondria-mediated apoptotic germ cell phagocytosis in Sertoli cells.

In the present study, a model of HS-induced apoptosis of germ cells in testis was developed and activation of GSK3α in SCs was used to investigate the regulation of GSK3α in phagocytic action. The obtained results suggest a novel role of GSK3α in the regulation of mitochondria-mediated apoptotic GCs phagocytosis in SCs. GSK3α activation inhibits mitochondrial fission via phosphorylation of Drp1 at Ser637. The schematic signaling pathway as suggested by these data is presented in Figure 7.

Paracrine signaling between GCs and SCs regulates the homeostasis between these intimately associated cells. SCs are phagocytic and help to remove the residual bodies while degenerating GCs by phagocytosis *in vivo*. Phagocytosis of dying GCs acts as a source of lipid availability to SCs for the germ cell development; lipid oxidation being is the predominant pathway for ATP production in SCs [2]. Mild testicular hyperthermia transiently increases lipid droplet accumulation and modifies both sphingolipid and glycerophospholipid acyl chains in the rat testis [17] and SCs *in vitro* [18]. The conducted experiments also showed that GSK3α activation led to lipid droplet accumulation in SCs both *in vivo* and *in vitro*. Spermatogenesis can be compromised by inactivation of genes involved in lipid metabolism, suggesting that an appropriate lipid metabolism is central for male reproduction [19]. Loss of key molecules in the lipid efflux such as ABCA1 or TXLNA results in the accumulation of lipid in SCs and impair of male fertility [4,20]. Under normal physiological conditions, mitochondrial β-oxidation is the dominant metabolic pathway for fatty acid oxidation. Immunohistochemistry showed that SCs in the seminiferous tubules contain a full set of mitochondrial fatty acid β-oxidation enzymes in relatively plentiful amounts among the cells in the testis; however, this is not so in spermatogenic cells [21]. These results suggest that a change of mitochondrial activity resulted in the accumulation of lipid droplets in SCs.

Recently, several studies indicated that GSK3 plays a pivotal role in the regulation of mitochondrial activity. In *Xenopus* oocytes, decrease of insulin signaling induced activation of GSK3 and mitochondrial ETC remodeling [22]. In contrast, the acute inhibition of GSK by inhibitor causes mitochondrial remodeling in the heart of mice [23]. Park et al. indicated that the mitochondrial membrane potential and is a key determinant of apoptotic cell clearance [6]. In the present study, GSK3α activation induced an alteration of mitochondrial morphology and decreased ΔΨm. These results suggest that GSK3α activation participates in the regulation of mitochondrial activity and resulted in the accumulation of lipid droplets and alteration of metabolism pattern in SCs.

Recently, Wang et al. demonstrated that the uptake of multiple apoptotic cells by macrophages requires Drp1-mediated mitochondrial fission [7]. Posttranslational modifications of Drp1 were central for its activity, including GTPase activity, mitochondrial distribution, and dimerization of Drp1 [24–26]. It has been demonstrated that phosphorylation of Drp1 at Ser637 could inhibit the translocation of Drp1 to mitochondria and thus promote mitochondrial fusion [12]. Our results suggest that GSK3α activation resulted in the downregulation of mitochondrial fission in TM4 cells accompanied by phosphorylation of Drp1 at Ser637. Moreover, the increase of phosphorylation at Ser637 induced by HS can be blocked by SB216763. Co-immunoprecipitation analysis indicated that GSK3α was detected to interact with Ser637 p-Drp1. These results provide compelling evidence that GSK3α activation is involved in Drp1-mediated mitochondrial fission. In summary, our data suggest that GSK3α activation decreased mitochondrial activity and mitochondrial fission.

In the present study, GSK3α in SCs could be inactivated by Ser21 phosphorylation. Activation of GSK3α in SCs resulted in damage of spermatogenesis while GSK3α inhibitor supplementation could restore this process. It was interesting that testis morphology, spermatogenesis, and sperm numbers appear normal in GSK3α deleted mouse [9]. Previous studies indicated that loss of GSK3α, either by pharmacologic inhibition or GSK3α gene deletion has the same function in age-related pathologies in mice [27]. These results suggest that inactivation of GSK3α in SCs is required for apoptotic germ cell phagocytosis.

Taken together, our results demonstrate that inactivation of GSK3α is required for mitochondria-mediated apoptotic GCs phagocytosis in SCs. GSK3α activation participates in decrease of mitochondrial activity and in altering the mitochondrial structure. Specifically, GSK3α activation inhibits mitochondrial fission via phosphorylation of Drp1 at Ser637. Changes of mitochondria resulted in the accumulation of lipid droplets and the alteration of the metabolic pattern of SCs. Together, the obtained results indicate that the normal occurrence of SC phagocytosis requires phosphorylation of GSK3α at Ser21. The results provide new insights for the understanding of the underlying mechanisms of phagocytic clearance of apoptotic spermatogenic cells by SCs during spermatogenesis.

## MATERIALS AND METHODS

### Ethics statement

The Institutional Animal Care and Use Committee (IACUC) of Nanjing Agricultural University approved all animal procedures. The “Guidelines on Ethical Treatment of Experimental Animals” (2006) No. 398 set by the Ministry of Science and Technology, China and the Regulation regarding the Management and Treatment of Experimental Animals” (2008) No. 45 set by the Jiangsu Provincial People’s Government, was strictly followed during the slaughter and sampling procedures.

### Reagents

Busulfan (B2635) was purchased from Sigma. Chemical Corporation (St. Louis, MO, USA). The terminal dexynucleotidyl transferase (TdT)-mediated dUTP nick end labeling (TUNEL) kits (C1086) were purchased from Beyotime Institute of Biotechnology (China). The JC-1 (5,5′,6,6′-tetrachloro-1,1′,3,3′tetraethylbenzymid-azolyl carbocyanine iodide) mitochondrial membrane potential (ΔΨm) kits were purchased from Nanjing KeyGen Development Co., Ltd. (China). The GSK3α inhibitor (SB216763, S1075) was purchased from Selleck Chemicals LLC (Houston, TX, USA). The ATP determination kits (S0026), triglyceride (TG) determination kits (A110) and lactic acid determination kits (A019) were purchased from Nanjing Jiancheng Bioengineering Institute (China).

### Animal treatment

Adult (8-10 weeks) male BALB/c mice were obtained from the Animal Research Center of Yangzhou University. The heat treatment experiment on the mice testis was performed three times, and a total of 24 mice were divided into three groups, with eight mice per group. After anesthesia with an intraperitoneal injection of sodium pentobarbital (40 mg/kg body weight), the tails and the scrotums containing the testis of heat-treated mice were immersed in a thermostatically controlled water bath at 42°C for 20 min, as previously described [28]. Animals were then dried and returned to their cages. Control animals were anesthetized and left for 20 min at room temperature. Mice were killed at 1 h, 3 h, or 24 h after hyperthermal exposure. In the busulfan treatment experiment, seven mice were treated with a single intra-peritoneal injection of 25 mg/kg busulfan. Busulfan was first dissolved in DMSO before equal volume of distilled water was added. Control animals (five mice) were treated with a single intra-peritoneal injection of DMSO. The testis tissue of each mouse was divided into two portions: one was fixed in Bouin’s solution and used for morphological examination, and the other was frozen in liquid nitrogen and prepared for Western blot analyses.

### Immunohistochemistry staining

Histological sections were treated with 3% (v/v) H_2_O_2_ to block endogenous peroxides and antigen retrieval was carried out in nitrate buffer at 95 °C for 20 min. Fetal bovine serum (FBS, 10% (v/v)) was used to block nonspecific staining. Then, the sections were respectively incubated with the following primary antibodies were used: rabbit anti-Phospho-GSK3α (Ser21) antibody (1:1000, Cell Signaling Technology, 9316, MA, USA), rabbit anti-GSK3α antibody (1:1000, Cell Signaling Technology, 4337, MA, USA), rabbit anti-Phospho-GSK3β (Ser9) antibody (1:1000, Cell Signaling Technology, 9336, MA, USA), rabbit anti-GSK3β antibody (1:1000, Cell Signaling Technology, 9315, MA, USA) at a dilution of 1:500 (v/v) overnight at 4 °C. The immunoreaction was achieved with the goat anti-rabbit antibody (Boster Bioengineering Co., Ltd., Wuhan, China) at a dilution of 1:200 (v/v) and developed with 3,3′-diaminobenzidine tetrahydro-chloride (DAB).

### Isolation of spermatogenic cells

The isolation of spermatogonia (primitive type A, mature type A and type B spermatogonia) performed by the STA-PUT method described by Bellve with minor modification [29,30]. Briefly, mice testes were harvested after euthanasia and digested with collagenase IV (1 mg/ml). The dispersed seminiferous tubules was washed with DMEM and centrifuged. The pellet was further digested with 0.25% Trypsin containing DNase I (1 mg/ml) and filtered to prepare a single-cell suspension. The single-cell suspension was loaded into a cell separation apparatus (ProScience Inc. Canada) and followed by 2–4% bovine serum albumin (BSA) gradient (2% BSA and 4% BSA in DMEM were loaded into the separation apparatus chamber). After 1.5-3 h of sedimentation, cell fractions were harvested. The purity of spermatogonia reaches about 90%.

### Protein extraction and western blot analysis

Testis and TM4 cell samples were homogenized in RIPA buffer (50 mM Tris-HCl pH 7.4, 150 mM NaCl, 1% NP40, 0.25% Na-deoxycholate, 1 mM PMSF, 1 mM sodium orthovanadate with Roche EDTA-free complete mini protease inhibitor cocktail, no. 11836170001). The protein concentration was measured with the BCA Protein Assay Kit (Pierce, Rockford, IL, USA)according to a previous publication [31]. Forty micrograms of protein extract were used for electrophoresis on a 15% or 10% SDS-PAGE gel. The following primary antibodies were used: rabbit anti-phospho-GSK3α (Ser21) antibody, rabbit anti-GSK3α antibody, rabbit anti-phospho-GSK3β (Ser9) antibody, rabbit anti-GSK3β antibody, rabbit anti-LC3 antibody (Novus Biologicals, NB100-2220, USA) and rabbit anti-DRP1 (phospho Ser637) antibody (1:1000, Abcam, ab193216, MA, USA). Protein loading controls for each experiment using rabbit anti-α-tubulin antibody (1:1000, Bioworld, bs1699, China). All the operations were carried out according to the recommended protocols provided by the manufacturers.

### TUNEL assay

Apoptosis assays were performed by TUNEL reactions using the Apoptotic kit. Then stained with Permanent Green substrate-chromogen and counterstained with eosin. The percentage of apoptotic, TUNEL-positive cells was expressed as the average number of apoptotic cells within 20 seminiferous tubes. A minimum of 100 seminiferous tubules were counted per testis (5 sections/testis) and at least 3 animals per genotype per age were assessed.

### Cell culture and treatment

The TM4 cell line used in this study was established by Mather in 1980 from primary cultures of SC isolated from 11 to 13 d old BALB/c mice [32]. They were seeded (1×10^5^ cells/dish) and cultured at 37°C for 24 h in Dulbecco’s modified Eagle’s medium (DMEM) (Gibco BRL, NY, USA) containing 10% fetal bovine serum, Penicillin/Streptomycin (100 mU/ml) in a saturated atmosphere of 5% CO_2_. The HS cells were removed into incubator at 42°C, and then returned to the incubator at 37°C.

### Phagocytosis assay

Before GCs were labeled with DAPI, GCs were harvested from control mice and induced to undergo apoptosis [5], which was confirmed by annexin V staining. Apoptotic germ cells were then labeled with DAPI for 5 min at room temperature in the dark, followed by washing and incubation with TM4 Sertoli cells. After 3 h of co-incubation, engulfed apoptotic germ cells were observed under the microscope or were subjected to flow cytometry analysis.

### Measurement of the mitochondrial membrane potential (ΔΨm)

TM4 cells were measured using a JC-1 fluorescent probe. Cells at 10×10^6^ cells/ml were incubated at 37°C or 42°C for 3 h before analysis by flow cytometry analysis according to previous publications [33]. Briefly, treated cells were centrifuged at 1000 g for 5 min and then stained with 2.5 μg/ml JC-1 for 15 min at 37°C. Then, cells were washed with ice-cold PBS twice, samples were analyzed via flow cytometry, and 10,000 events were acquired on the flowcytometer. JC-1 emissions from excitation at the 488 nm were collected at 525 nm (JC-1 green) and 585 nm (JC-1 red). Gates, including the final gate for dye excluding cells were subjectively set based on the flow cytometry images; however, within each experiment, the same gate settings were used to determine dye exclusion cohort percentile changes that resulted from experimental maneuvers.

### Transmission electron microscopy (TEM) observation of mitochondria and autophagosome

TM4 cells with or without SB216763 were incubated at 37°C or 42°C for 3 h and were fixed with 2% glutaraldehyde, post-fixed with 1% osmium tetroxide, and embedded in resin. Ultrathin sections were cut and stained with uranyl acetate and lead citrate. The sperm ultrastructure was determined with a transmission electron microscope (Hitachi H-7650, Hitachi Technologies, Tokyo, Japan).

### Structured illumination microscopy (SIM)

TM4 cells were stained with 200 nmol/L MitoTracker Red CMXRos (Molecular Probes, USA) for 45 min at 38.5°C under 5% CO_2_, followed by washing in TCM-199 twice. Cells were then fixed with 4% paraformaldehyde in PBS for 15 min at room temperature. After three washes with TCM-199 for 20 min, the stained cells were viewed under an N-SIM microscope (Nikon, Tokyo, Japan).

### Co-immunoprecipitation

Co-immunoprecipitation was performed as previously described with minor modifications. Five hundred micrograms of total protein were precleared with 40 μL of protein A/G plus beads for 1 h at 4°C and then, centrifuged at 1,000×g for 5 min. The supernatants were incubated with 4 μg GSK3α antibodies and rotated overnight at 4°C. Thereafter, 40 μL of agarose beads were incubated with the protein-antibody complexes for 2 h at 4°C. After centrifugation, the agarose beads were washed and the immunoprecipitated proteins were run on 7.5% SDS-polyacrylamide gel for western blot analysis.

### ORO staining

The lipid droplets were visualized by ORO staining. Briefly, Sertoli cells were fixed with 10% formalin for 40 min, and stained with ORO solution (Sigma, ORO saturated solution in isopropanol: water, 3:2) for 15 min. The background staining was removed by washing the cells with 70% alcohol for 5 s. The lipid droplets in Sertoli cells were analyzed under a microscope (IX71, Olympus, Japan).

### Determination of triglyceride, ATP and lactic acid content

The triglyceride levels were determined using TG Color GPO/PAP kit based on comparison with a concurrent standard curve. The ATP content measurements were obtained using an ATP determination kit based on comparison with a concurrent standard curve. The TM4 cell culture media were collected, then lactate content in supernatant was measured by using lactate assay kit following the manufacturer’s instruction.

### Statistical analysis

All data are presented as mean ± SEM and were analyzed using independent samples *t*-test and Two-way ANOVA followed by LSD post hoc test with SPSS 16.0 for windows. The differences were considered statistically significant when *P* <0.05.
